# Influence of Pokémon GO on Physical Activity and Psychosocial Well-Being in Children and Adolescents: Systematic Review

**DOI:** 10.2196/49019

**Published:** 2023-11-13

**Authors:** Haiyan Liang, Xi Wang, Ruopeng An

**Affiliations:** 1 School of Sport Business Guangzhou Sport University Guangzhou China; 2 Brown School Washington University in St Louis St Louis, MO United States

**Keywords:** Pokémon GO, artificial intelligence, physical activity, psychosocial well-being, children, adolescent

## Abstract

**Background:**

Pokémon GO, an augmented reality game with widespread popularity, can potentially influence players’ physical activity (PA) levels and psychosocial well-being.

**Objective:**

This review aims to systematically examine the scientific evidence regarding the impact of Pokémon GO on PA and psychosocial well-being in children and adolescents.

**Methods:**

Using the PRISMA (Preferred Reporting Items for Systematic Reviews and Meta-Analyses) guidelines and the GRADE (Grading of Recommendations, Assessment, Development, and Evaluations) framework, we conducted keyword and reference searches in the PubMed, CINAHL, Web of Science, and Scopus databases. We performed title and abstract screening, full-text review, evidence synthesis, and identified research gaps.

**Results:**

Our review included 10 studies that explored the effect of Pokémon GO on PA or psychosocial well-being among children and adolescents. These studies used diverse designs across multiple countries and regions. Pokémon GO use measures encompassed frequency, experience, adherence, and motivation. PA assessment methods ranged from self-reported questionnaires to technology-based evaluations and qualitative approaches. Psychosocial well-being measures included emotional intelligence, personal well-being, self-control, emotionality, and sociability. In general, the estimated impact of Pokémon GO on PA was positive, with gaming elements and engagement correlating with increased PA levels. However, the effect on psychosocial well-being presented mixed results, with positive associations for sociability but a complex relationship involving well-being and internet gaming disorder. The limitations of these studies comprised the absence of randomized controlled trials, heterogeneity in study designs and outcome measures, and potential confounding bias.

**Conclusions:**

Overall, Pokémon GO tends to positively affect PA levels, while the impact on psychosocial well-being remains complex and requires further investigation. Future research should investigate the mechanisms connecting Pokémon GO use with PA and psychosocial well-being and the potential risks of excessive gameplay. These findings can help inform public health interventions to harness gaming technologies for promoting PA and enhancing well-being among the younger generation.

**Trial Registration:**

PROSPERO International Prospective Register of Systematic Reviews CRD42023412032; https://www.crd.york.ac.uk/PROSPERO/display_record.php?RecordID=412032

## Introduction

Physical activity (PA) and psychosocial well-being are essential components of a healthy lifestyle, particularly for children and adolescents, as they contribute to overall physical and mental health, cognitive development, academic performance, and social skills [1-3]. In children and adolescents, PA refers to any form of exercise or bodily movement that promotes cardiovascular health, muscle strength, flexibility, and bone density, thereby facilitating growth, development, and the establishment of lifelong fitness habits [4]. Psychosocial well-being refers to the interrelation of psychological factors and social elements in children and adolescents that contribute to their overall mental and emotional health [5]. This encompasses aspects of cognitive performance, emotional intelligence, and the balance of recreational activities [5]. Cognitive performance can be understood through indicators such as memory, attention, concentration, and creativity [6]. Emotional intelligence pertains to factors such as self-awareness, emotional regulation, and interpersonal effectiveness, with attributes such as well-being, self-control, emotionality, and sociability [7]. Balanced psychosocial well-being also considers the potential risks of excessive gaming, such as addiction or compulsive behaviors, and how these might impact the broader aspects of a young individual’s life, such as their relationships and academic performance [8].

In recent years, the growing availability and use of mobile health apps have presented unique opportunities to promote PA and an active lifestyle on a broader scale [9]. Pokémon GO (Niantic Inc), an augmented reality game, has captured the interest of millions of users worldwide and holds the potential to promote PA and enhance psychosocial well-being among children and adolescents. While not explicitly designed for this purpose, Pokémon GO shares similarities with exergames, a genre of active video games that promotes energy expenditure, weight loss, and overall health [1]. GPS-based games such as Pokémon GO incorporate mobile gameplay and exploration, provide an engaging alternative to traditional PA, and foster social interaction and outdoor exploration [2]. Despite the mixed evidence on the effectiveness of exergames and activity-based video games in promoting PA [10], Pokémon GO represents a promising avenue for public health efforts to encourage an active lifestyle and improved well-being among young populations.

Pokémon GO’s unique features and characteristics make it a potentially effective tool for promoting PA and psychosocial well-being among children and adolescents. The game’s incorporation of augmented reality, exploration, and social interaction aligns with the fundamental principles of health behavior and well-being theories [11]. For instance, the self-determination theory (SDT) [12] posits that individuals are more likely to engage in activities that satisfy their innate psychological needs for competence, autonomy, and relatedness. Pokémon GO’s gameplay encourages players to develop skills, make choices, and connect with others to pursue digital creatures, fostering an environment conducive to intrinsic motivation and sustained engagement [13]. Furthermore, the social cognitive theory [14] emphasizes the role of observational learning and reinforcement in shaping behavior [15]. Pokémon GO facilitates positive reinforcement through rewards, such as catching rare Pokémon or leveling up, which can encourage continued PA [16]. The game’s social features, such as cooperative raids and player versus player battles, provide opportunities for children and adolescents to interact, learn from one another, and develop social connections, potentially enhancing their psychosocial well-being [17]. However, it is crucial to consider the potential risks associated with excessive or addictive gameplay [18,19]. The dual systems model of adolescent risk-taking highlights the imbalance between the development of reward-driven impulses and cognitive control systems during adolescence. This imbalance may predispose children and adolescents to excessive gameplay, resulting in negative consequences such as reduced time for other essential activities, social isolation, or even physical injuries.

While previous reviews have shed light on the impact of Pokémon GO on PA and psychosocial well-being, they have primarily focused on adult populations. For instance, Baranowski et al [1] found modest increases in PA among older adults, while Khamzina et al [3] reported a statistically significant but clinically modest increase in daily steps taken by Pokémon GO players. Furthermore, Lee et al [2] suggested that the game could improve walking behavior and psychological and social well-being. To our knowledge, no review has specifically examined the effects of Pokémon GO on children and adolescents.

Considering the unique developmental stages and health behavior formation processes of younger populations, our systematic review sought to address this gap by focusing exclusively on children and adolescents [2]. We concurrently examined PA and psychosocial well-being, as PA has been consistently shown to positively influence psychosocial well-being in this age group, leading to improved self-esteem, social skills, and mental health outcomes [20]. One potential avenue for Pokémon GO to affect psychosocial well-being may be through increased PA. By offering the most recent review in this area, we aimed to inform public health interventions and policy making, harnessing the potential of popular gaming technologies to promote PA and enhance well-being among the young generation.

## Methods

### Overview

The review was carried out following the PRISMA (Preferred Reporting Items for Systematic Reviews and Meta-Analyses) guidelines (Multimedia Appendix 1) [21,22]. The review was registered in PROSPERO (CRD42023412032).

### Study Selection Criteria

Studies that met all of the following criteria were included in the review: (1) study design: experimental studies (eg, randomized controlled trials [RCTs], pre-post interventions, and cross-over trials), observational studies (eg, cross-sectional and prospective cohort studies), and qualitative studies (eg, interviews and focus groups); (2) study subjects: children and adolescents (younger than 18 years old); (3) outcomes: PA measures (eg, daily steps and exercise duration) or psychosocial well-being measures (eg, self-esteem and social support); (4) article type: original, empirical, and peer-reviewed journal publications; (5) time window of search: from the inception of an electronic bibliographic database to March 20, 2023; and (6) language: articles written in English.

Studies were excluded from the review if they met any of the following criteria: (1) studies with a focus on outcomes unrelated to PA (eg, diet and sleep) and psychosocial well-being; (2) studies focusing on adults; (3) non-English language articles; and (4) letters, editorials, study or review protocols, case reports, or review articles.

### Search Strategy

A keyword search was performed in 4 electronic bibliographic databases such as PubMed, CINAHL, Web of Science, and Scopus. The search algorithm included “Pokemon GO” or “Pokémon GO.” Multimedia Appendix 2 presents the search algorithm used in each database. The search algorithm was intentionally made simple without incorporating other terms related to PA, psychological well-being, or children and adolescents. This arrangement ensured we minimized the risk of missing relevant studies during the comprehensive literature search stage. Two coauthors (HL and XW) of this review independently screened the title and abstract for the articles found through the keyword search, obtained potentially relevant articles, and reviewed their full texts. The interrater agreement between these 2 authors (HL and XW) was evaluated using Cohen κ (κ=0.89). Disagreements were settled through discussion.

A reference list search (ie, backward reference search) and a cited reference search (ie, forward reference search) were conducted based on the full-text articles identified from the keyword search that met the study selection criteria. Articles identified from the backward and forward reference search were further screened and evaluated using the same study selection criteria. Such procedures were repeated on all newly identified articles until no additional relevant article was found.

### Data Extraction and Synthesis

Using a standardized data extraction form, the following methodological and outcome variables were collected from each study: author, publication year, country or region, study design, intervention design, sample size, age range, the proportion of female participants, measures of Pokémon GO use, measures of PA, measures of psychosocial well-being, estimated effect of Pokémon GO on PA, and estimated effect of Pokémon GO on psychosocial well-being. Two coauthors (HL and XW) independently conducted the data extraction, and discrepancies were resolved through discussion with a third coauthor (RA). Heterogeneous exposure and outcome measures prevented meta-analysis, so we narratively summarized the common themes and findings of the included studies.

### Study Quality Assessment

GRADE (Grading of Recommendations, Assessment, Development, and Evaluations) is a framework for developing and presenting evidence summaries and provides a systematic approach to making clinical practice recommendations [23]. GRADE evaluates and assigns each study 1 of the 4 levels of evidence: very low, low, moderate, and high. RCTs start at high quality or evidence; due to residual confounding, observational studies start at low quality or evidence. The level of quality or evidence for a study is increased or decreased during the evaluation process using the GRADE criteria concerning the risk of bias, imprecision, inconsistency, indirectness, and publication bias [24].

## Results

### Study Selection

**Figure 1:**
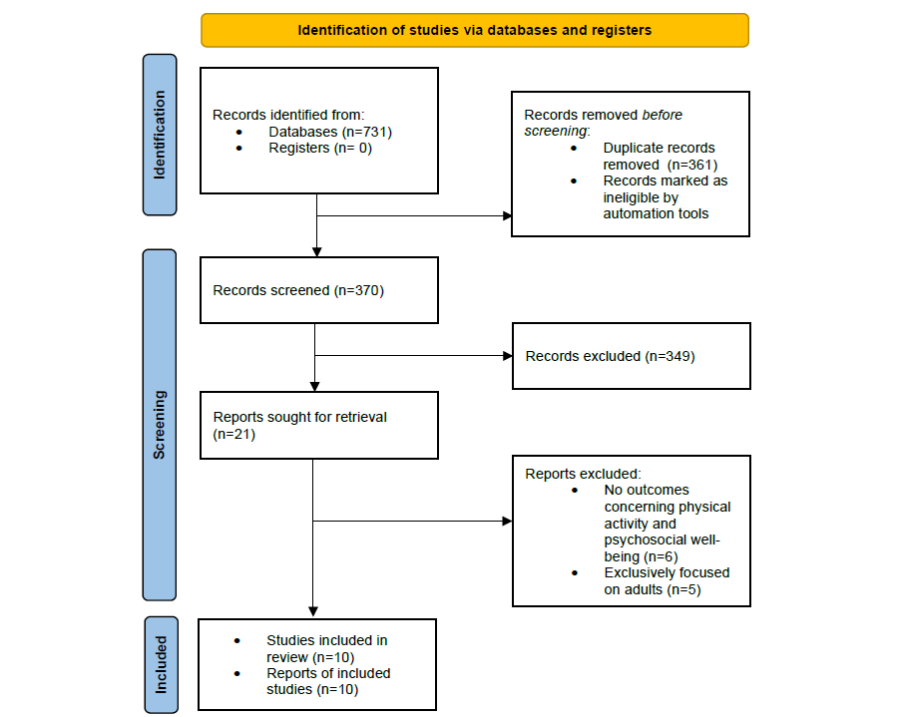
PRISMA (Preferred Reporting Items for Systematic Reviews and Meta-Analyses) flow diagram.

Figure 1 shows the study selection flowchart. We identified 731 articles through keyword and reference searches, including 96 from PubMed, 156 from CINAHL, 221 from Web of Science, and 258 from Scopus. After removing duplicates, 370 articles underwent title and abstract screening, of which 349 were excluded. The remaining 21 articles were reviewed in the full text against the study selection criteria. Of these, 11 were excluded—6 reported no outcomes concerning PA and psychosocial well-being and the remaining 5 exclusively focused on adults. Therefore, a final pool of 10 articles was included in the review [25-34].

### Study Characteristics

Table 1 summarizes the essential characteristics of the 10 studies included in the review. All studies were published within the last 5 years, with 4 studies in 2018 [27,29,30,34], 3 in 2019 [28,32,33], and 3 in 2022 [25,26,31]. The studies were conducted in various countries and regions, including Sweden [30], Hong Kong [29], the United States [25,27], Spain [26,34], Taiwan [32,33], Peru [28], and Indonesia [31]. Different study designs were used, including qualitative (interview and focus group) [30,31], cross-sectional [27-29,33], prospective cohort [25], and pre-post studies [26,31,32,34]. The intervention duration ranged from 6 to 10 weeks. Sample sizes ranged from 13 to 944 participants. The age of participants also varied across studies, with some focusing on younger children (ages 5-12 y) and others on adolescents (ages 13-18 y). Two studies included both children or adolescents and adults [27,29]. The percentage of female participants ranged from 23% (7/31) to 56.2% (86/153) across the included studies.

**Table 1:** Essential characteristics of the studies included in the review^a^.

Study ID	Authors (year)	Country or region	Study design	Intervention design	Sample size, n	Age range (y)	Female, n (%)	GRADE quality^b^
1	Lindqvist et al [30] (2018)	Sweden	Qualitative (focus group)	N/A^c^	13	7-12	4 (30.8)	Low
2	Ma et al [29] (2018)	Hong Kong	Cross-sectional	N/A	210 (25 of them were 13-17 years old)	13-17	71 (33.8）	Low
3	Militello et al [27] (2018)	The United States	Cross-sectional	N/A	160 parents and 31 children	5-17	7 (23)	Low
4	Ruiz-Ariza et al [34] (2018)	Spain	Pre-post (with a control group)	The control group did not play Pokémon GO, but the experiment group played for 7 weeks	190	12-15	94 (49.5)	Moderate
5	Cheng [33] (2019)	Taiwan	Cross-sectional (postevaluation)	Playing Pokémon GO for 6 weeks	466	13-15	192 (41.2)	Low
6	Hsieh and Chen [32] (2019)	Taiwan	Pre-post (with a control group)	The control group did not play Pokémon GO, but the experiment group played for 10 weeks	123	11-13	63 (51.2)	Moderate
7	Mejia et al [28] (2019)	Peru	Cross-sectional	N/A	944	13-16	450 (47.7)	Low
8	Jumareng et al [31] (2022)	Indonesia	Pre-post (with a control group) Qualitative (interview)	The control group did not play Pokémon GO, but the experiment group played for 7 weeks	94	15-18	44 (46.8)	Moderate
9	Martínez-López et al [26] (2022)	Spain	Pre-post (with a control group)	The control group did not play Pokémon GO, but the experiment group played for 8 weeks	164	12-15	81 (49.4)	Moderate
10	Wang et al [25] (2022)	The United States	Prospective cohort	N/A	153	9-11	86 (56.2)	Moderate

^a^The column on intervention design has missing values for the nonintervention studies.

^b^GRADE: Grading of Recommendations, Assessment, Development, and Evaluations.

^c^N/A: not applicable.

### Measures of Pokémon GO Use

The measures of Pokémon GO use adopted by the included studies are summarized in Table 2. These include frequency of play, such as times played in a month and app installation status [29,30]. Some studies also considered the players’ experience, differentiating between current players and those who played in the past [25,27,31]. The level of adherence and the amount of PA were frequently measured using accumulated points, the number of Pokémon captured, distance traveled in kilometers, or daily game time in minutes [26,28,32,34]. Furthermore, motivation for playing was assessed, as well as categorizing use into levels such as no use, playing a little (less than 2 hours per day), and playing a lot (over 2 hours per day) [28,33].

**Table 2:** Measures and estimated impact of Pokémon GO.

Study ID	Authors (year)	Measures of Pokémon GO use	Measures of PA^a^	Measures of psychosocial well-being	Estimated effect of Pokémon GO on PA	Estimated effect of Pokémon GO on psychosocial well-being
1	Lindqvist et al [30] (2018)	Times played in a month	Focus group followed by qualitative latent content analysis based on Graneheim and Lundman [35] (2004)	N/A^b^	Gaming elements such as cooperating and exploring are positively associated with players’ PA	N/A
2	Ma et al [29] (2018)	Pokémon GO app installation status	Daily walking and running distances: measured by iPhone “health” app	N/A	Based on the multilevel modeling (using MLwiN V.3.0), installing the Pokémon GO app is positively associated with daily walking and running distances (post: mean 6.26, SD 2.45 vs pre: mean 5.30, SD 2.12; F1,418=33.825; *P*<.001) Pokémon GO is positively associated with daily walking and running distances: multilevel models (coefficient 1=0.085, *P*<.001; coefficient 2=0.084, *P*<.001)	N/A
3	Militello et al [27] (2018)	Pokémon GO play experience: current player or played in the past	LSI^c^ of parents: Godin-Shepard leisure-time PA questionnaire Parental influence on children’s PA: general parenting support, active parents, past activity, and guiding support	N/A	Based on the Spearman rank correlation and *t* tests, playing Pokémon GO is positively associated with parents LSI (n=160; post: mean 48.04, SD 25.96 vs pre: mean 38.25, SD 25.84; *P*<.001) Parents’ LSI is positively associated with children’s PA (n=31; pre-LSI ρ=0.503, *P*=.004; post-LSI ρ=0.476, *P*=.007)	N/A
4	Ruiz-Ariza et al [34] (2018)	Level of adherence and the amount of PA: accumulated points, number of Pokémons captured, distance traveled in kilometers, and daily game time in minutes	N/A	Emotional intelligence: well-being, self-control, emotionality, and sociability TEIQue-SF^d^ by Petrides [36] (2009)	N/A	Based on the ANCOVA^e^ tests, playing Pokémon GO is positively associated with the sociability score (post: mean 4.76, SD 1.06 vs pre: mean 4.40, SD 0.71; *P*=.04) The sociability score of the intervention group is 9.87% (CG^f^: n=103; IG^g^: n=87) higher than the control group (intervention: mean 4.76, SD 1.06 vs control: mean 4.40, SD 0.71; *P*=.003)
5	Cheng [33] (2019)	Motivation for playing: a modified 20-item questionnaire by Korgaonkar and Wolin [37] (1999) and Liu et al [38] (2010)	N/A	Temperament: a 10-item questionnaire based on Rothbart and Hwang [39] Addiction: IGD-20^h^ test (threshold>71) Well-being: Well-being Questionnaire by Kurtz and Welch [33]	N/A	Based on SEM^i^ (using AMOS^j^ version 22.0; IBM Corp), motivation for playing Pokémon GO is positively associated with IGD-20 (coefficient=0.46; *P*<.001) and well-being (coefficient=0.17; *P*<.010)
6	Hsieh and Chen [32] (2019)	Level of adherence and the amount of PA: accumulated points, number of Pokémons captured, distance traveled in kilometers, and daily game time in minutes	N/A	Emotional intelligence: well-being, self-control, and sociability Emotional intelligence: TEIQue-SF by Petrides [36] (2009)	N/A	Based on the ANCOVA tests, playing Pokémon GO positively correlates with the sociability score (post: mean 4.68, SD 0.621 vs pre: mean 4.32, SD 0.563; *P*=.03) Sociability score of the intervention group is 7.69% (CG: n=62; IG: n=61) higher than that of the control group (intervention: mean 4.68, SD 0.621 vs control: mean 4.32, SD 0.563; *P*=.003)
7	Mejia et al [28] (2019)	Levels of Pokémon GO use: No use. Played a little (<2 hours per day). Played a lot (>2 hours per day)	N/A	Internet or video game addiction: MULTICAGE CAD-4 test	N/A	Based on the generalized linear models (using STATA; StataCorp), the level of Pokémon GO use is positively associated with video game addiction. (PR^k^=1.33, 95% CI 1.07-1.65)
8	Jumareng et al [31] (2022)	Pokémon GO play status: classified into players, ex-players, and nonplayers Exposure to the Pokémon GO program	PA level measured by IPAQ^l^ In-depth interview followed by thematic analysis	N/A	Based on the *t* tests, playing Pokémon GO is positively associated with PA in all three groups: (1) players (post: mean 2246.8, SD 742.4 vs pre: mean 1881.1, SD 456.1; *P*<.001); (2) ex-players (post: mean 2517.6, SD 650.7 vs pre: mean 1733.5, SD 423.2; *P*=.001); and (3) nonplayers (post: mean 2506.9, SD 666.5 vs pre: mean 1740.5, SD 687.3; *P*=.001)	N/A
9	Martínez-López et al [26] (2022)	Level of adherence and the amount of PA: accumulated points, number of Pokémons captured, distance traveled in kilometers, and daily game time in minutes	Physical fitness: CRF^m^, MS^n^, S/A^o^ Body weight status: BMI, body fat, waist-hip index MVPA^p^ questionnaire by Prochaska [40] (2001)	N/A	Based on the ANCOVA test, playing Pokémon GO is positively associated with physical fitness CRF (post: mean 6.23, SD 1.43 vs pre: mean 5.53, SD 1.75; *P*<.006) CRF of the intervention group is 22.2% (CG: n=86; IG: n=78) higher than the control group (mean 6.23, SD 1.43 vs mean 5.09, SD 1.87; *P*=.009)	N/A
10	Wang et al [25] (2022)	Pokémon GO play status classified into current players and current nonplayers	MVPA measured by the GT3X model accelerometer	N/A	Based on the *t* tests, playing Pokémon GO is not associated with MVPA (intervention: mean 27.57, SD 21.94 vs control: mean 28.52, SD 19.17; t144=0.15; *P*=.88)	N/A

^a^PA: physical activity.

^b^N/A: not applicable.

^c^LSI: Leisure Score Index.

^d^TEIQue-SF: Trait and Emotional Intelligence Questionnaire Short Form.

^e^ANCOVA: analysis of covariance.

^f^CG: control group.

^g^IG: intervention group.

^h^IGD-20: Internet Gaming Disorder Test-20.

^i^SEM: structural equation modeling.

^j^AMOS 22.0: Analysis of Moment Structures 22.0.

^k^PR: prevalence ratio.

^l^IPAQ: International Physical Activity Questionnaire.

^m^CRF: cardiorespiratory fitness.

^n^MS: muscular strength.

^o^S/A: speed/agility.

^p^MVPA: moderate to vigorous physical activity.

### Measures of PA

Among the 10 studies included in the review, 6 [25-27,29-31] reported measures of PA encompassing various assessment methods (Table 2). Common measures include self-reported questionnaires such as the Godin-Shephard Leisure-Time Physical Activity Questionnaire or the International Physical Activity Questionnaire [27,31]. Some studies used technology-based assessments such as the iPhone “health” app to measure daily walking and running distances and accelerometers to measure moderate-to-vigorous intensity physical activity [25,29]. Some studies focused on the parental influence on children’s PA and general parenting support [27]. In addition, some studies used qualitative approaches such as focus groups (followed by latent content analysis) and in-depth interviews (followed by thematic analysis) [30,31]. Physical fitness measures such as cardiorespiratory fitness, muscular strength, speed or agility, body weight status, BMI, body fat, or waist-hip index were also used [26].

### Measures of Psychosocial Well-Being

Among the 10 studies included in the review, 4 reported measures of psychosocial well-being (Table 2) [28,32-34]. Common measures across these studies encompassed emotional intelligence, personal well-being, self-control, emotionality, and sociability, often assessed using the Trait Emotional Intelligence Questionnaire-Short Form [32,34]. Additionally, 1 study [33] focused on temperament, using a 10-item questionnaire based on Rothbart and Hwang [39], and well-being, assessed by the Well-being Questionnaire by Kurtz and Welch [33]. Two studies also investigated internet or video game addiction, where 1 used the Internet Gaming Disorder-20 and the other used MULTICAGE CAD-4 tests [28,33].

### Estimated Effect of Pokémon GO on PA

Among the 6 studies that reported PA-related outcomes [25-27,29-31], the overall estimated effect of Pokémon GO on PA measures was generally positive (Table 2) [25,31]. The findings suggest that gaming elements such as cooperation and exploration are positively associated with players’ PA levels [30]. Moreover, installing the Pokémon GO app and playing the game was positively associated with daily walking and running distances, leisure-time PA, and physical fitness measures [26,27,29]. In some cases, parents’ involvement in Pokémon GO was also positively associated with their children’s PA levels [27].

The duration of increased PA varied across the studies [25-27,29-31]. Of the 6 studies, 4 reported significant increases in PA associated with playing Pokemon GO [26,27,29,31]. The increased PA duration aligned with active gameplay duration in most studies. Specifically, Jumareng et al [31] found increased PA over a 7-week gameplay period. Martínez-López et al [26] showed increased PA after 8 weeks of gameplay. Wang et al [25] and Lindqvist et al [30] did not specify the exact gameplay duration but found higher PA among active players compared to nonplayers cross-sectionally. Only 1 study, Militello et al [27], examined whether increased PA was sustained beyond active gameplay. They surveyed players at 1, 6, and 12 months after the release of Pokemon GO and found significant increases in mild and moderate PA at all 3 time points, suggesting the PA benefits persisted beyond active gameplay [27]. In summary, most studies demonstrated increased PA during active gameplay for 1 week to 2 months. Only 1 study reported that PA increases sustained for at least 12 months after downloading the game [30]. Further research is needed to determine whether playing Pokemon GO yields lasting PA improvements after gameplay ceases.

### Estimated Effect of Pokémon GO on Psychosocial Well-Being

Among the 4 studies that reported psychosocial well-being-related outcomes, the overall estimated effect of Pokémon GO on psychosocial well-being is mixed (Table 2) [28,32-34]. On the one hand, playing Pokémon GO has been found to be positively associated with sociability scores in 2 studies, with intervention groups showing higher sociability scores compared to control groups [32,34]. On the other hand, motivation for playing Pokémon GO was positively associated with internet gaming disorder and well-being, indicating a more complex relationship [33]. Additionally, 1 study reported a positive association between the level of Pokémon GO use and video game addiction [28].

### Study Quality Assessment

We assessed the quality of the studies included in the review using the GRADE framework (Table 1) [24]. Five studies were rated “moderate” [25,26,31,32], while the remaining 5 were rated “low” in study quality [27-30,33]. The primary reason for a “low” rating was the cross-sectional or qualitative study design, which is prone to confounding bias. On the other hand, the “moderate” rating was primarily due to studies adopting a pre-post or longitudinal study design, which offers more robust causal inferences than cross-sectional or qualitative study designs. However, these studies are still subject to confounding issues due to the lack of randomization.

## Discussion

### Principal Findings

This study systematically reviewed the existing scientific evidence to discern the effects of Pokémon GO, a globally popular augmented reality game, on PA and psychosocial well-being in children and adolescents. By systematically searching 4 databases, this review scrutinized 10 divergent studies from various countries and regions. The assessments and resultant analysis of these studies revealed that engagement with Pokémon GO is generally correlated with increased levels of PA among the youth, which supports the game’s potential use as a public health intervention tool to combat physical inactivity. Nonetheless, the game’s impact on psychosocial well-being was multifaceted, demonstrating favorable outcomes in sociability [32-34] but illustrating a nuanced interplay between well-being and internet gaming disorder [28,33]. This inconsistency underscores the imperative for more granular research to unweave the intricate relationships between augmented reality gaming, PA, and various dimensions of psychosocial well-being in young populations and to formulate interventions that maximize benefits and mitigate risks associated with gameplay.

### Comparison With Prior Work

The findings of this systematic review, which focused on children and adolescents, align with previous reviews that primarily investigated the effects of Pokémon GO on adults. Baranowski et al [1] found that although PA increases from playing Pokémon GO appeared to be small and short-lived among young adults, there may be mental and social health benefits [1]. Similarly, Khamzina et al [3] reported a statistically significant but clinically modest increase in the daily steps taken among game players, emphasizing the challenge of retaining active engagement once the novelty wears off [3]. In our review, Pokémon GO was found to have a generally positive effect on PA measures, with gaming elements and engagement positively associated with PA levels. Lee et al [2] found that Pokémon GO players had greater PA than nonplayers and experienced improved social interactions, mood, selective attention, and concentration [2]. The game also promoted meaningful improvements in walking behavior and psychological and social well-being [2]. Our review discovered similar findings, with positive associations between playing Pokémon GO and sociability scores among children and adolescents but also indications of a more complex relationship involving both well-being and internet gaming disorder.

The underlying mechanisms linking Pokémon GO use to increased PA and improved psychosocial well-being among children and adolescents could be understood through health behavior and psychosocial theories. The social cognitive theory posits that personal factors, environmental factors, and human behavior influence behavior change [14,15]. Pokémon GO, as an augmented reality game, creates a unique environment that combines digital elements with real-world exploration, which can intrinsically motivate children and adolescents to be more physically active [13]. Furthermore, the game encourages social interactions by enabling players to collaborate, compete, and communicate with others, thereby fostering a sense of community and social support [30]. These social aspects may improve psychosocial well-being by satisfying the need for relatedness, as described in the SDT [12]. The SDT also emphasizes the importance of autonomy and competence in promoting intrinsic motivation [41]. Pokémon GO allows players to make choices, set personal goals, and develop skills, which may further enhance their engagement in PA and contribute to improved well-being.

On the other hand, excessive and addictive Pokémon GO gameplay among children and adolescents can have various negative consequences. From a behavioral and psychological standpoint, excessive gameplay can be attributed to operant conditioning [28]. Players become conditioned to seek immediate rewards and gratification through in-game achievements, ultimately leading to addictive behavior [42]. The time spent on Pokémon GO may displace other essential activities such as sleep, academic work, and social interactions, potentially impacting overall well-being and development. Moreover, studies have reported safety concerns related to Pokémon GO gameplay, such as traffic violations, trespassing, and other risky behaviors [43]. These concerns may stem from a lack of situational awareness and impaired decision-making as players become immersed in the game, consistent with the Limited Capacity Model of Motivated Mediated Message Processing [44]. The model suggests that when cognitive resources are devoted to gameplay, individuals may have a reduced capacity to process and respond to real-world environmental cues, increasing the likelihood of engaging in risky behaviors [44]. Therefore, it is crucial to promote responsible gameplay and parental supervision, emphasizing the importance of balancing digital activities with other essential aspects of life and ensuring that Pokémon GO and similar games do not adversely affect the health and safety of children and adolescents.

Multiple factors informed our review’s exclusive focus on Pokémon GO rather than other GPS and location-based games. First, Pokémon GO, with its augmented reality interface, stands out from many other location-based games due to its blend of real-world interaction and digital gameplay [45]. This distinct feature uniquely positions the game to potentially foster both PA and social interaction in an immersive environment, a combination not commonly found in most GPS-based games [46]. Second, the immense popularity and global reach of Pokémon GO, with millions of users spanning various age demographics, offer a more extensive and diverse data pool than lesser-known games [47]. Significantly, Pokémon GO’s allure is not limited solely to children and adolescents. It uniquely engages family units, often with parents or older siblings participating alongside younger players, creating a rich dynamic that integrates recreational activity with family bonding [48]. Furthermore, the game’s significant media coverage and its sparking of global phenomena have led to abundant research specifically focused on Pokémon GO, allowing for a more comprehensive and robust analysis [49]. While previous reviews explored Pokémon GO’s impact on adults, a discernable gap existed in literature specific to younger populations [50]. Given the game’s potential implications for child and adolescent health, especially considering their developmental stages and the formation of health behaviors, it was imperative to address this gap [51]. By concentrating on Pokémon GO, we could delve deeper into its intricate impacts on PA and psychosocial well-being among children and adolescents, providing valuable insights for potential interventions and policies targeted at this age group.

### Limitations and Strengths

The systematic review and the included studies exhibit several limitations that should be considered when interpreting the findings. First, none of the included studies adopted an RCT design. This limitation raises concerns about the internal validity of the findings. Second, the relatively small number of studies included in the review may limit the comprehensiveness and generalizability of the results. Additionally, the diverse measures used for PA and psychosocial well-being across the included studies prevented a meta-analysis, thus limiting the review to a narrative synthesis. Furthermore, the small or medium sample sizes in the included studies were not nationally or regionally representative, which may affect the generalizability of the findings to the broader child and adolescent population. Another limitation is the heterogeneity of the studies in terms of geographical location, setting, and study design, further complicating the generalization of the findings. In addition, no study evaluated PA and psychosocial well-being simultaneously, making it difficult to understand how these 2 factors may interact and influence the overall impact of Pokémon GO on children and adolescents.

The existing body of literature assessing Pokémon GO’s impact on psychosocial well-being in children and adolescents has primarily focused on direct associations. However, 1 area that merits further exploration is the potential mediating role of PA in this relationship. Pokémon GO’s influence on well-being might be multifaceted, with direct and indirect effects channeled through enhanced PA. While the direct effects of the game on well-being are noteworthy, it is imperative to consider that some observed benefits might be, in part, attributed to increased PA, a recognized enhancer of psychosocial health. Future studies could offer a more comprehensive understanding by incorporating measures of PA. This would allow for a clearer distinction between the direct effects of gameplay and the potential mediating influence of associated physical activities. Such an approach promises a more nuanced appreciation of how digital games such as Pokémon GO can shape the psychosocial health of young individuals.

The assessment of PA and psychosocial well-being in the included studies presents methodological challenges. The diversity of tools, from objective measures such as the GT3X model accelerometer [25] to qualitative methods such as focus groups [30] and subjective measures such as the iPhone health app [29], introduces potential inconsistencies in capturing PA behaviors. Similarly, the range of instruments for evaluating psychosocial well-being, including the Trait Emotional Intelligence Questionnaire-Short Form [32,34] and the Internet Gaming Disorder-20 test [33], suggests potential discrepancies in operational definitions and assessments. While these tools are grounded in empirical research, their varied use can complicate cross-study comparisons and may not provide a comprehensive understanding of the impacts of Pokémon GO.

### Future Directions

Future research would benefit from greater standardization in measurement tools to ensure consistent and comprehensive evaluations of PA and psychosocial well-being. For assessing psychosocial well-being among children and adolescents, recognized measures such as the Strengths and Difficulties Questionnaire [52], Pediatric Quality of Life Inventory [53], and Kidscreen Instruments [54] provide comprehensive insights. Regarding PA, objective tools such as ActiGraph accelerometers [55] and the SenseWear Armband [56] offer reliable data capture, while structured recalls such as the Pediatric Physical Activity Recall [57] can present a more structured self-reporting method. Using these validated measures in research can enhance the robustness of findings and support cross-study comparisons.

### Conclusions

This systematic review investigated the effects of Pokémon GO on PA and psychosocial well-being among children and adolescents, incorporating 10 diverse studies conducted in various countries and regions. The main findings indicate that Pokémon GO use generally has a positive effect on PA levels, while the impact on psychosocial well-being is more mixed, revealing both positive associations with sociability scores and a complex relationship involving well-being and internet gaming disorder. Despite the limitations of the included studies and the review itself, such as the lack of RCT designs, small sample sizes, and heterogeneity in measurements and populations, the findings contribute to understanding how Pokémon GO influences the health and well-being of children and adolescents. Future research should address these limitations and explore the potential interaction between PA and psychosocial well-being. Additionally, researchers should investigate the underlying mechanisms and pathways that contribute to both positive and negative outcomes associated with Pokémon GO use, which could inform the development of more effective and engaging interventions to promote health and well-being in young populations.

## Appendix

Multimedia Appendix 1 PRISMA (Preferred Reporting Items for Systematic Reviews and Meta-Analyses) checklist.

Multimedia Appendix 2 Search algorithm in databases.

## Abbreviations

GRADE: Grading of Recommendations, Assessment, Development, and Evaluations
PA: physical activity
PRISMA: Preferred Reporting Items for Systematic Reviews and Meta-Analyses
RCT: randomized controlled trial
SDT: self-determination theory
