# Supplementary material for: Influence of Pokémon GO on Physical Activity and Psychosocial Well-Being in Children and Adolescents: Systematic Review
Source: J Med Internet Res. 2023 Nov 13;25:e49019. doi: 10.2196/49019 (PMC10682915; doi:10.2196/49019)
Supplement: Multimedia Appendix 2 [file jmir_v25i1e49019_app2.docx]

| Database | Search algorithm |
| --- | --- |
| MEDLINE, CINAHL Plus (searched in EBSCO) | ((TI “Pokemon Go” OR “Pokémon Go”) or (AB “Pokemon GO” OR “Pokémon Go”)) and (LA “English”) |
| Web of Science | TI = (“Pokemon Go” OR “Pokémon Go”) or AB = (“Pokemon Go” OR “Pokémon Go”) and Article (Document Types) and English (Languages) and Article (Document Types) |
| Scopus | TITLE-ABS-KEY (“Pokemon Go” OR “Pokémon Go”) AND (LIMIT-TO (DOCTYPE , “ar”) ) AND (LIMIT-TO (LANGUAGE , “English”) ) |
| PubMed | “Pokemon Go”[TIAB] OR “Pokémon Go”[TIAB] |
